# CD38: T Cell Immuno-Metabolic Modulator

**DOI:** 10.3390/cells9071716

**Published:** 2020-07-17

**Authors:** Anwesha Kar, Shikhar Mehrotra, Shilpak Chatterjee

**Affiliations:** 1 Cancer Biology and Inflammatory Disorder Division, CSIR-Indian Institute of Chemical Biology, Kolkata 700032, India; anwesha@csiriicb.res.in; 2 Department of Surgery, Medical University of South Carolina, Charleston, SC 29425, USA

**Keywords:** CD38, NAD^+^, T cell differentiation, metabolism, chromatin remodeling

## Abstract

Activation and subsequent differentiation of T cells following antigenic stimulation are triggered by highly coordinated signaling events that lead to instilling cells with a discrete metabolic and transcriptional feature. Compelling studies indicate that intracellular nicotinamide adenine dinucleotide (NAD^+^) levels have profound influence on diverse signaling and metabolic pathways of T cells, and hence dictate their functional fate. CD38, a major mammalian NAD^+^ glycohydrolase (NADase), expresses on T cells following activation and appears to be an essential modulator of intracellular NAD^+^ levels. The enzymatic activity of CD38 in the process of generating the second messenger cADPR utilizes intracellular NAD^+,^ and thus limits its availability to different NAD^+^ consuming enzymes (PARP, ART, and sirtuins) inside the cells. The present review discusses how the CD38-NAD^+^ axis affects T cell activation and differentiation through interfering with their signaling and metabolic processes. We also describe the pivotal role of the CD38-NAD^+^ axis in influencing the chromatin remodeling and rewiring T cell response. Overall, this review emphasizes the crucial contribution of the CD38^−^NAD^+^ axis in altering T cell response in various pathophysiological conditions.

## 1. Introduction

T cells have evolved to mount protective response against invading pathogens and cancers, while maintaining tolerance to self-antigens [1,2]. This is particularly governed by the intricate balance between the activation signals (signals required for T cell activation and clonal proliferation) and inhibitory signals (signals that dampen the T cell effector response) [3]. While optimum activation signals are required for T cells to resolve infections and tumor burden, inhibitory signals restrain T cells from mounting immune response against self-antigens.

Activation of T cells following encountering of “non-self” foreign pathogens or “self” tumor antigens occurs in a highly coordinated fashion [4]. To be fully activated, T cells require three distinctive receptor mediated activation signals, “signal 1” which is delivered through TCR upon recognition of cognate antigen presented by MHC [4,5], “signal 2” through co-stimulatory molecules like CD28, GITR, OX-40, etc. [3], and “signal 3” through cytokine-cytokine receptor interaction [6]. Failure in engaging any of the three signals results in transducing sub-optimal strength signal for T cell activation, which finally leads to T cell anergy [7,8].

Similar to activation, the magnitude and durability of the T cell response are also kept in tight check by various cellular mechanisms to avoid the collateral damage of host tissues due to exaggerated inflammation. It is known that the expression of various co-inhibitory receptors, such as PD1, CTLA4, Lag3, Tim3, on T cells serves as a brake to counterbalance the activation signals initiated by the stimulatory receptors [3,9]. Therefore, co-inhibitory receptors seem to play a pivotal role in T cell homeostasis by controlling T cell effector response and thus have major implications in diverse disease pathobiology, including cancer and autoimmunity [10].

In addition to various co-stimulatory and co-inhibitory T cell receptors, ectonucleotidases that regulate the extracellular concentration of nucleotides, are also considered pivotal in modulating T cell response [11,12]. It has been shown that ectonucleotidases like CD39 and CD73 can promote an immunosuppressive microenvironment in various diseases like cancer, autoimmunity, and allergy through generation of adenosine by sequential cleavage of extracellular ATP to AMP and AMP to adenosine [11]. By doing this conversion, CD39 and CD73 impinge on purinergic signaling in T cells by limiting the availability of purinergic mediator ATP, and hence mitigate the pro-inflammatory response of T cells [12]. This demonstrates the emerging role of ectonucletidases as key regulator in determining the generation of inflammatory vs. immunosuppressive T cell response.

In recent years, CD38, another critical ectonucleotidase has gained prominence as an important regulator of T cell activation and function [13,14]. CD38 is a multifunctional transmembrane ectoenzyme that belongs to nicotinamide adenine dinucleotide (NAD^+^) glycohydrolase/adenosine 5′-diphosphate-ribosyl cyclase gene family. The enzymatic activity of CD38 not only catalyzes the cyclization of NAD^+^ to cyclic ADP-ribose (cADPR), but also hydrolyzes cADPR to form ADP-ribose (ADPR) [15]. Interestingly, it has been shown that a small amount of NAD^+^ gets cyclized by CD38 to produce cADPR, while the majority is hydrolyzed to ADPR [16]. This observation led to the proposition that the major enzymatic activity of CD38 is NAD^+^ glycohydrolase (NADase), but not ADP-ribosyl cyclase. In addition to NAD^+^, CD38 has also been shown to hydrolyze nicotinamide adenine dinucleotide phosphate (NADP) into nicotinic acid adenine dinucleotide phosphate (NAADP) via a base-exchange reaction [17]. However, the reaction requires an acidic pH and high (millimolar) concentration of nicotinic acid, the conditions can only be attained in vitro but hardly possible in vivo [17]. In fact, this notion was further supported by the observation that shRNA mediated knockdown of CD38 in Jurkat T cells had no effect in altering the intracellular concentration of NAADP, suggesting the dispensable role of CD38 in generating NAADP [18].

Numerous studies suggest that cADPR generated by the enzymatic action of CD38 acts as a second messenger for intracellular Ca^2+^ mobilization in several cells [19,20]. This indicates a plausible involvement of CD38 in regulating T cell activation [14,21], given the unequivocal role of Ca^2+^ signaling in triggering T cell activation. In fact, it has been reported that the expression of CD38 accompanies T cell activation and predominantly localizes to the immune synapse in close contact with T cell receptor (TCR) [22]. Moreover, the NAD^+^ glycohydrolase (NADase) activity of CD38 which determines the intracellular level of NAD^+^ [16], a principal metabolite regulating diverse biochemical and cellular processes further evinces the pivotal role of CD38 in regulating T cell functionality. Here in, we will focus on how CD38 is involved in regulating T cell-mediated immunity.

## 2. CD38-NAD^+^ Axis in Health and Diseases

CD38 was discovered as a cell surface marker present on the thymocytes and activated T cell surface and initially termed as T10 [23,24]. The enzymatic activities of CD38 generating ADPR and cADPR were described by Berthelier et al. and De Flora et al. [25,26]. A decade later, this molecule drew attention after Edward Chini and colleagues unearthed the role of CD38 as a major NAD^+^ catabolizing enzyme having a number of pathophysiological implications in aging, infection, and tumorigenesis [15].

High expression of CD38 has often been found to be associated with several hematological malignancies [27,28]. For example, the pathogenic role of CD38 have been implicated in multiple myeloma (MM), where tumor cells exhibit high surface expression of CD38 [29,30]. Likewise, CD38 expression is reported in other hematological tumors including B cell-chronic lymphocytic leukemia, acute myeloid leukemia, acute lymphocyte leukemia, and acute promyelocytic leukemia [31]. Owing to high CD38 expression, therapeutic interventions targeting CD38 are being devised for various hematological malignancies. Recently, monoclonal antibody targeting CD38 has been approved by FDA for the treatment of patients with refractory MM [27,28,32,33]. Conversely to hematological tumors, malignant cells from solid tumors do not express CD38. However, emerging studies are indicating that immune cells of both lymphoid and myeloid origin present at solid tumor sites exhibit high cell surface expression of CD38, which negatively correlates with the prognosis of the disease [13,34,35].

In contrast to causal attribution of CD38 in hematological malignancies many intriguing pieces of evidence suggest that CD38 is an essential component that serves to combat various infections by triggering innate immune response. A study in mice with *Listeria monocytogenes* infection has shown that upregulation of CD38 on neutrophils and macrophages is essential for their recruitment to the site of infection and efficient pathogen clearance [36]. In accord with this observation, an earlier study in C57BL/6 mice with *Mycobacterium avium* infection also implicated the role of CD38 in mounting protective immune response against the pathogen [37]. Mechanistically, CD38 has been shown to facilitate signaling pathways that lead to the production of pro-inflammatory cytokines from DC and macrophages [38,39,40,41], which appears to be instrumental in restraining infectious burden. Recent findings also indicate that the expression of CD38 can act as a negative regulator of immune cell function. In multiple myeloma, CD38 is implicated in promoting more aggressive immunosuppressive MDSCs and Treg [42]. A similar observation was also reported in the cases of esophageal and colorectal cancer (CRC) patients, where expression of CD38 potentiates the suppressive function of MDSCs and hence is associated with poor survival of patients [35,43]. These studies thus demonstrate that apart from acting as an adhesion molecule through interaction with CD31 on endothelial cells, CD38 could also tinker with the cellular events leading to distinctive functional outcome by immune cells.

Although, much efforts have been made to elucidate the role of CD38 in B cell malignancies and innate immune cells, its relative contribution in modulating T cell response is still limiting. Earlier studies reported the expression of CD38 on human early T cell precursors and on CD4^+^CD8^+^ double positive thymocytes [44]. In contrast, mature T cells have low level of CD38 but its expression is enhanced by various lymphocytes activators [45,46]. In fact, a number of studies from Fabio Malavasi’s group reported that in vitro cross-linking of CD38 with specific monoclonal antibodies on human T cells are capable of inducing its activation, proliferation and cytokine secretion through triggering different signaling events [47,48,49]. Owing to these facts, CD38 has long been considered as the activation marker for T cells. Most recently, a transient increase in the frequency of both CD4^+^ and CD8^+^ CD38^+^HLA-DR^+^ T cells was observed in the blood sample from patient with COVID-19 during the viral clearance phase (day 7–9) [50]. This population (CD4^+^ and CD8^+^ CD38^+^HLA-DR^+^ T cells) has been shown to be positively corelated with the improved outcome of the patient [50]. However, CD38 has also been characterized as a marker of terminally exhausted T cells, which are refractory to the PD1 blockade mediated functional rejuvenation [51,52]. In agreement with this observation, a study from our group also reported that expression of CD38 caused metabolic aberration and compromised anti-tumor response by T cells [13]. These intriguing evidences suggest a complex role of CD38 in regulating T cell response through intervening multiple cellular and molecular pathways.

## 3. CD38 Mediated Signaling in Activated T Cells

The importance of CD38 in regulating T cell function is increasingly appreciated owing to their multifunctional enzymatic activity (both NADase and ADP-ribosyl cyclase), which can deplete intracellular NAD^+^ level and generates key signaling mediator, cADPR in T cells concomitantly [14]. However, in lymphocytes, CD38 is present on the plasma membrane in a type II conformation, with its catalytic domain exposed extracellularly [53,54]. This observation aroused the question of how CD38 metabolizes intracellular NAD^+^ and generates cADPR, an intracellular second messenger, while its catalytic domain faces outside. In a study by Zhao et al., this issue was addressed and they found that CD38 could be positioned in the plasma membrane in a type III orientation, with its C-terminal catalytic domain would be facing the cytoplasm [55]. Therefore, the type III conformation of CD38 appears to be crucial for its intracellular signaling activity and hence could be important for mediating the cADPR induced intracellular Ca^2+^ signaling.

Ca^2+^ signaling is known to play a vital role in T cell activation and differentiation [56]. The engagement of TCR with its cognate antigen in the context of MHC results in an increase in intracellular Ca^2+^ concentration through store-operated calcium entry (SOCE) and activation of calcium release-activated calcium (CRAC) channels [56]. In T cells, the surge in Ca^2+^ concentration following TCR stimulation is predominantly triggered by 1,4,5-inositol triphosphate (IP3), which has been shown to mobilize Ca^2+^ from ER lumen through binding with IP3 receptors (IP3Rs) on ER membrane [57,58,59]. Although IP3Rs were found to be important for antigen triggered Ca^2+^ release in T cells, it could not explain prolonged (>1 h) Ca^2+^ signaling by CRAC as IP3 levels returned to near basal levels within 10 min following TCR stimulation [60,61,62]. This led to the possibility of other mechanisms operating in parallel to the IP3-IP3R axis in mobilizing Ca^2+^ from ER following TCR stimulation. Studies by Geuse et al. had shown that ryanodine receptors (RyRs), another Ca^2+^ release channel also contributed to Ca^2+^ release from ER lumen through binding to the second messenger cADPR in a TCR stimulation dependent way. Using high-performance liquid chromatography analysis, they showed that stimulation of the T-cell receptor/CD3 (TCR/CD3) complex resulted in the activation of a soluble ADP-ribosyl cyclase and a sustained increase in intracellular levels of cADPR. Increased cADPR significantly and specifically stimulated type-3 ryanodine receptor, indicating a direct modulatory effect of cADPR on Ca^2+^ channel opening [63,64] and hence T cell activation and proliferation. Furthermore, the CD38 mediated cADPR production could indirectly induce increase in intracellular Ca^2+^ level in T cells by inhibition of the sarcoendoplasmic reticulum Ca^2+^ ATPase (SERCA), which facilitates calcium entry into ER from cytosol [56,65]. These studies together provide direct evidences that T cell activation, proliferation, and differentiation could be regulated by the CD38 dependent cADPR-RyR axis owing to its ability to modulate intracellular Ca^2+^ signaling.

**Figure 1:**
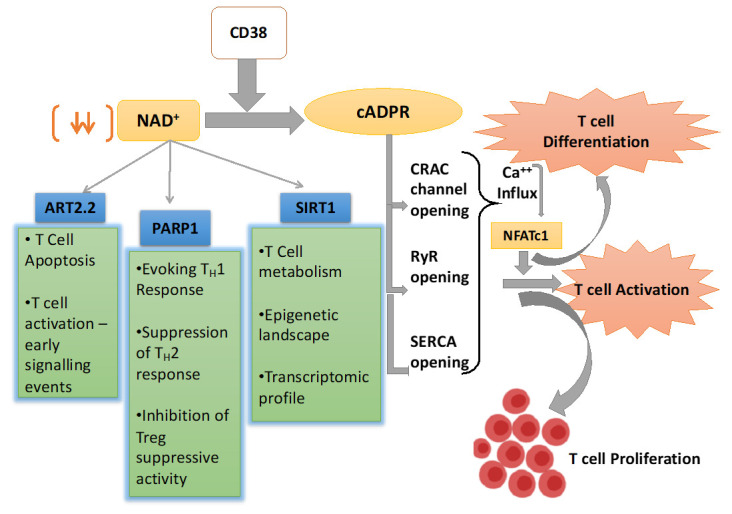
Schematic representation of nicotinamide adenine dinucleotide (NAD^+^) utilizing pathways inside the T cell and their overall effect on T cell response. CD38 is the major mammalian NAD^+^ glycohydrolase (NADase) which metabolizes NAD^+^ and generates cyclic ADP-ribose (cADPR), which promotes T cell activation and proliferations through facilitating Ca^2+^ signaling. CD38 expression also depletes NAD^+^ level and hence affect the enzymatic activity of different NAD^+^ consuming enzymes like Sirt1, PARP1, and ART2.2, which play pivotal role in T cell fate determination.

In addition to mobilizing Ca^2+^ from ER, the role of CD38 induced cADRP-RyR axis in regulating T cell functionality has also been reported. It was shown that splenocytes from CD38 deficient mice with *M. avium* infection were skewed towards Th2 type and secreted lower IFN-γ, which correlated with their compromised ability to limit mycobacterial burden [37]. This is in agreement with the earlier observation showing that human T cells upon CD38 ligation in vitro secreted several pro-inflammatory cytokines, including IFN-γ, IL-6, GM-CSF, and IL-10 [48]. It can be conceivable that the reported effect of CD38 in regulating cytokines production by T cells could be mediated through the activation of NFATc1 by cADPR-RyR axis induced Ca^2+^ signaling [66]. It has been shown that activation-induced Ca^2+^ influx in T cells results in nuclear localization of NFATc1 that drives the expression of several genes associated with T cell functionality, including the expression of various cytokines genes [67]. Chromatin immunoprecipitation (ChIP) assay has revealed that NFATc1 has a putative binding site in the regulatory region of IL-2, IL-4, and IFN-γ in T cells, and hence can control their expression [67]. Therefore, it seems that CD38 can act as an upstream regulator of intracellular Ca^2+^ signaling that could activate NFATc1 and hence determine the functionality of the T cells. Apart from regulating Ca^2+^ signaling, an association between activation-induced expression of CD38 and triggering of MAPK pathway has also been demonstrated where PTK, CD3-z/ZAP-70/PLC-g1 played a significant role [68]. Considering these findings, CD38-cADPR-Ca^2+^axis in T cells must be explored in more detail to further unravel the underlying mechanisms driving T cell functionality (Figure 1).

## 4. CD38-NAD^+^ Axis in Regulating T cell Fate and Function

CD38 has been identified as a critical modulator of NAD^+^ metabolism owing to its NADase activity [15,16]. NAD^+^ is a crucial cellular metabolite being, directly and indirectly, involved in a plethora of signaling pathways. Intracellular NAD^+^ level dependent regulation of various signaling cascades is shown to be mediated through two important enzymes, Poly (ADP-ribose) polymerase (PARP) and sirtuins (Sirt), which utilize NAD^+^ as substrate [69,70,71]. PARP is a family of proteins involved in several cellular processes such as DNA repair, genomic stability, and programmed cell death [72]. Sirt (Sirt1-6) are a class of proteins that possess either mono-ADP-ribosyltransferase, or deacylase activity, including deacetylase, desuccinylase, demalonylase, demyristoylase, and depalmitoylase activity [73]. Several studies reveal that overexpression of CD38 leads to the depletion of intracellular NAD^+^ levels and thus has a profound influence on the activity of the NAD^+^ consuming enzymes (PARP and Sirt), which regulate cellular homeostasis [16].

Alongside the PARPs and Sirt, whose activity is principally governed by the availability of the intracellular NAD^+^, there exists another class of NAD^+^ consuming enzyme named as ADP-ribosyl transferases or ARTs that act as extracellular NAD^+^ sensors. There are two major isoforms of ARTs—ART2.1 and ART2.2, which are reported to play a critical role in T cell activation and fate determination [74,75,76,77]. In addition, directly interfering early events of TCR signaling through producing cADPR, the NADase activity of CD38 could also have a profound influence on various aspects of T cell activation and differentiation. In the next few sections, we will be elaborating, how CD38 dependent modulation of NAD^+^ levels affect different cellular events in T cells, which in turn dictate the functional and phenotypic outcome of T cells (Figure 1).

### 4.1. NAD^+^ Dependent Mono-ADP-Ribosyl Transferases (ARTs) in T Cells

Mono-ADP-ribosyl transferases or ARTs are the class of GPI-anchored ecto-enzymes that catalyzes the covalent attachment of ADP-ribose moiety of NAD^+^ to arginine residues on target proteins [72]. Four human (ARTC1, 3, 4, 5) and six mice (ARTC1, 2.1, 2.2, 3, 4, 5) ART proteins have been characterized till now, and their tissue-specific distribution has also been analyzed [78]. The role of ARTs in T cells was ascertained by the observation that treatment with ART inhibitors or ART substrate NAD^+^ had a profound effect on mouse T cell proliferation, cytotoxicity, homing, and TCR clustering [74,75,77]. These effects have mainly been attributed to the ART mediated ADP-ribosylation of several T cell surface proteins, including LEF-1 and CD8 [76]. In addition to interfering with T cell activation and cytotoxicity, ART is also implicated in NAD^+^-induced cell death (NICD) of T cells [79]. It is reported that ART2.2 dependent ADP-ribosylation of P2RX7 represents an alternative pathway of triggering P2RX7 activation on T cells in the presence of extracellular NAD^+^ [78,79]. Further, this event leads to Ca^2+^/Na^2+^ influx and efflux of K^+^ ions, shedding of CD62L, externalization of phosphatidyl serine (PS), cell shrinkage, and ultimately cell death if P2RX7 activation prolonged [79,80,81]. The notion of extracellular NAD^+^-ART axis mediated T cell death is further strengthened by the observation that ART2 knockout mice are resistant to NAD-induced apoptosis [82]. However, it has also been shown that T cells from C57BL/6 mice despite having high ART2, are relatively resistant to the effect of NAD^+^, suggesting that other factors in addition to ART2.2 are required for NICD of T cells [83]. Contrary to naïve T cells, activated T cells are resistant to NICD—an effect which could be in part due to proteolytic cleavage of ART2.2 from the activated T cell surface [83,84]. The reduced enzymatic activity of ART2.2 on activated T cells due to the expression of CD38 could also be critical in this context, as the expression of CD38 on activated T cells has shown to render T cells NICD resistant [85].

Apart from inducing apoptosis in response to extracellular NAD^+^, ARTs also regulate T cells activation and differentiation. Immuno-precipitation assays using mouse T cell lines (YAC1 and CTLL2) have confirmed CD25 to be a target for ART2.2 mediated ADP-ribosylation, which occurs at R35 residue of the IL-2 binding site and hence inhibits IL2 signaling via STAT5 [86]. Therefore, it seems that the competition between CD38 and ART2.2 for the occupancy of NAD^+^ might dictate the early events of T cell activation as ART2 could attenuate T cell activation by interfering with the IL2 signaling, whereas CD38 dependent cADPR mediated signaling events could promote T cell activation.

### 4.2. NAD^+^ Dependent Poly-ADP-Ribose Polymerases in T Cells

Similar to ARTs, which catalyze extracellular ADP-ribosylation, PARPs are the enzymes responsible for Poly-ADP-ribosylation of nuclear/cytoplasmic proteins [69,72]. Amongst various PARPs, PARP1 accounts for the majority of the poly-(ADPribose) polymer synthesis and functions as a DNA nick sensor binding to single and double strand breaks [87]. Compelling studies are suggesting that PARP-1 can also be activated through different mechanisms other than DNA damage and appear to be important in regulating T cell activation and differentiation [88]. A study by Vlador et al. reported that PARP1 dependent poly-ADP ribosylation negatively regulated the transcriptional activity of NFATc1 and NFATc2 in human T cell line (Jurkat) as this modification expedited nuclear export of NFAT, possibly through priming/facilitating their phosphorylation [89]. In contrast to this report, Olabisi et al. demonstrated that in mouse T cells direct binding and ADP ribosylation of NFAT by PARP1 positively regulated the transcriptional activity of PARP1 through increasing its avidity towards DNA and hence facilitated IL2 production and T cell activation [90]. The study further revealed that PARP1 and NFAT both individually and synergistically increased IL2 production in mouse T cells upon anti-CD3/anti-CD28 stimulation in a dose-dependent manner, an effect which is lost in PARP1^−/−^ T cells [90]. In addition to regulating IL2 and IL4, PARP1 has been shown to cause sustained expression of various inflammatory cytokines, including TNFα, IL1, IL6, etc. [91]. The mechanistic underpinning of PARP1 induced inflammation is the activation of NF-kB [92,93]. It has been shown that PARylation is vital to retain p65 NF-kB in the nucleolus by decreasing its interaction with Crm1 [94].

In mouse T cells, PARP1 has been shown to regulate multiple gene expression, including genes encoding for cytokines and chemokines [95]. Interestingly, PARP1 deficiency in T cells tipped the balance towards Th1 differentiation while demeaned Th2 response [95]. PARP1 mediated regulation of the Th2 response could be explained by the fact that inhibition of PARP1 facilitates the calpain-mediated degradation of STAT6, which is required for IL4 signaling [96]. In regulatory T cells (Treg), strikingly, it has been reported that activation of PARP1 seems to destabilize FoxP3 and reduce the suppressive activity of Treg [97]. In PARP-1^−/−^ Tregs, Foxp3 has been shown to bind at the conserved non-coding DNA sequence 2 (CNS2) at the *foxp3* gene, a region important in maintaining Foxp3 gene expression in Tregs [97]. This report followed the earlier study showing that activation of PARP1 negatively affects the differentiation of Tregs [98].

PARP1 has also been shown to play a crucial role in CD8 T cell homeostasis. It has been reported that oxidative stress-induced apoptosis of CD8 T cells is mediated through PARP1 activation [99]; possibly the mechanism is being exploited by tumor cells to evade the anti-tumor immune response. These studies together point to the fact that PARP1 activity in T cells has a profound influence in regulating T cell activation, function, and differentiation. Thus, PARP mediated pathways have emerged as a new CD38 regulated downstream pathway that could be therapeutically exploited to modulate T cell functions in various disease conditions.

### 4.3. NAD^+^-Sirt1 Dependent Regulation of T Cell Function

Sirt are the members of Class III histone deacetylase (HDAC) with the unique feature of using NAD^+^ as a co-factor [73]. Mammals are known to express seven Sirt (Sirt1-Sirt7) with different subcellular distribution: Sirt1, Sirt6, and Sirt7 are predominantly found in the nucleolus, Sirt2 in the cytoplasm, and Sirt3, Sirt4, and Sirt5 in the mitochondria [73]. It is known that Sirt has widespread implications in different pathophysiological conditions like inflammation, autoimmunity, cardiometabolic diseases, and cancers [100].

#### 4.3.1. Regulation of T Cell Effector Function

Amongst different Sirt, Sirt1 has emerged as an essential regulator of determining T cell fates and effector function. T cells isolated from Sirt1 knockout (Sirt1KO) mice exhibited hyper-proliferation and augmented IL2 production, features of hyperactivated T cells, which led to the development of experimental autoimmune encephalomyelitis (EAE) in Sirt1KO mice [101,102]. Subsequent studies underscoring mechanistic insights revealed that expression of Sirt1 could attenuate the activity of different transcription factors, which have shown to be indispensable for dictating T cell functions and phenotypes [103]. It has been reported that Sirt1 could negatively regulate T cell activation by inhibiting AP-1 transcriptional activity [104], which binds to the IL2 promoter region and stimulates IL2 production in T cells [105]. Sirt1 mediated negative regulation of T cell activation has also been shown to be mediated by the inhibition of *Bclaf1* transcriptional activity [106]. It has been reported that Sirt1 could form a complex with Rel-A (member of NFκβ family) and p300 (a histone acetyl transferase) at the *Bclaf1* promoter region and triggered deacetylation of histone 3 lysine 56 residue (H3K56) at *Bclaf1* promoter, led to the suppression of the transcriptional activity of *Bclaf1* and hence production of IL2 in T cells [106]. The notion of Sirt1 mediated negative regulation of T cell effector response was further strengthened by the observation that Sirt1 KO mouse showed the development of primary SLE like symptoms characterized by deposition of immune complexes within liver and kidneys, some even exhibited diabetes insipidus-like autoimmune disorder after 2 years of age [107].

#### 4.3.2. Th2 Response

In addition to regulating T cell effector function, Sirt1 exerts a profound effect on the differentiation of T cell subsets. Pharmacological inhibition of Sirt1 in ovalbumin induced murine model of asthma has shown to suppress Th2 differentiation, resulting in reduced airway inflammation in mice [108]. Although, the effect was found to be mediated by compromised ability of lung DC to stimulate Th2 response in presence of Sirt1 inhibitors (sirtinol and cambinol), T cell intrinsic attenuation of Sirt1 in facilitating its differentiation to Th2 cannot be ruled out [108].

#### 4.3.3. Th17 Response

The involvement of Sirt1 in regulating Th17 response is, however, intriguing, with apparently controversial reports having been published. A study by Lim et al. reported that Sirt1 potentiated Th17 response by increasing the transcriptional activity of RORγt, the signature transcription factor for Th17 generation [109]. The study further reported that T cell-specific genetic ablation of Sirt1 in a mouse model of multiple sclerosis significantly ameliorated the disease condition [109]. The observation is supported by our recent study showing that Sirt1 deacetylase activity is required for the differentiation of Th1/17 hybrid T cells capable of producing IFNγ and IL-17 simultaneously and mount robust anti-tumor response [13]. In contrast to these reports, study by Limagne et al. by using Sirt1 pharmacological agonists resveratrol and metformin showed that induction of Sirt1 impedes Th17 generation in mice through deacetylation of STAT3, a post-translational modification that repress nuclear translocation and subsequent STAT3 mediated transcriptional activation of *RORγT* [110]. In agreement with this observation, Wang et al. showed that in vivo activation of Sirt1 by NAD^+^ treatment delayed the pathogenesis of EAE in C57BL/6 mice by impeding the differentiation of both Th1 and Th17 response through downregulation of NF-κB [111]. Therefore, more explicit insights are required to unravel the contribution of the Sirt1 axis in regulating Th17 response in varied disease scenarios.

#### 4.3.4. Treg Differentiation

Protein deacetylase activity of Sirt1 has been shown to inversely correlates with the suppressive activity of Treg, owing to its crucial role in regulating the stability and transcriptional activity of FoxP3, the signature transcription factor of Treg [112,113]. It has been shown that Sirt1 can bind and deacetylate FoxP3 [112,113], which in turn increases the rate of FoxP3 turnover as deacetylation renders FoxP3 to K48 poly-ubiquitinylated and subjected to proteasomal degradation [114,115]. Further studies reveal that Sirt1 mediated deacetylation of three lysine residues (K31, K262, and K267) in murine FoxP3 not only impedes its stability but also hampers the ability of FoxP3 to bind to DNA and exert transcriptional activity [116]. In addition to post-translational control, targeting Sirt1 has also shown to increase transcript levels of FoxP3 mRNA [113]. Sirt1 mediated transcriptional regulation of FoxP3 could be mediated in part by affecting the acetylation of p65 (RelA) [113], which together with c-Rel promoted the formation of a FoxP3-specific enhanceosome, and hence increased the expression of FoxP3 [117]. As a result, a strategy to increase Treg by targeting Sirt1 has been exploited in various preclinical models of autoimmunity and inflammatory disorders and found that Sirt1 could be used as an essential target to improve Treg numbers and their suppressive activity [100,113,118].

## 5. CD38-NAD^+^ Axis in T Cell Immuno-Metabolism

Given the role of CD38 in modulating intracellular NAD^+^ levels, its involvement in regulating the metabolic commitment of T cells is becoming apparent [13,14]. Several studies are shedding light on CD38^−^NAD^+^-Sirt1 axis as an important metabolic checkpoint having enormous contribution in varied aspects of cellular energy metabolisms, including glycolysis, oxidative phosphorylation (OXPHOS), glutaminolysis, which are inherently associated with dictating T cell functional fate [13,119,120].

A recent study from our group illustrated that the ablation of the surface expression of the CD38 in CD4 T cells exhibited intrinsically higher levels of NAD^+^, which contributed to the rewiring of metabolic commitment and altered mitochondrial dynamics that renders T cells more effective in terms of anti-tumor immunity [13]. The study further reported that although targeting CD38 concomitantly enhanced Sirt1 activity, but the metabolic changes were independent of Sirt1, as Sirt1 deficiency had minimal effect on metabolic changes observed in CD38^−/−^ CD4 T cells [13]. This indicated the possibility that other CD38 dependent pathways could play an essential role in this process. One possibility could be the involvement of other Sirt like Sirt3, as it is reported that CD38 promotes the age-related decline of NAD^+^ which causes mitochondrial dysfunction and reduced OXPHOS in Sirt3 dependent manner [121]. The metabolic dysfunctionality observed in the CD38 expressing tumor-infiltrating CD4 T cells [13] could also be mediated by the loss of anti-oxidant potential of T cells. Studies published by our group and others have shown that expression of CD38 inversely regulates the anti-oxidant potential of cells, and loss of CD38 in CD4 T cells significantly increased the expression of various anti-oxidant genes including *Trx1, Trx2*, *Sod1, Sod2,* and *Nrf2* [13,122]. Recently, it has been shown that elevated ROS generation in T cells as a result of diminished anti-oxidant (glutathione) level, led to compromised activation of mTOR and reduced expression of NFAT and Myc, an important transcription factor drives glutaminolysis in T cells [123]. Therefore, further investigation in this direction is needed to delineate the Sirt1 independent role of CD38 in regulating the metabolic features of T cells.

**Figure 2:**
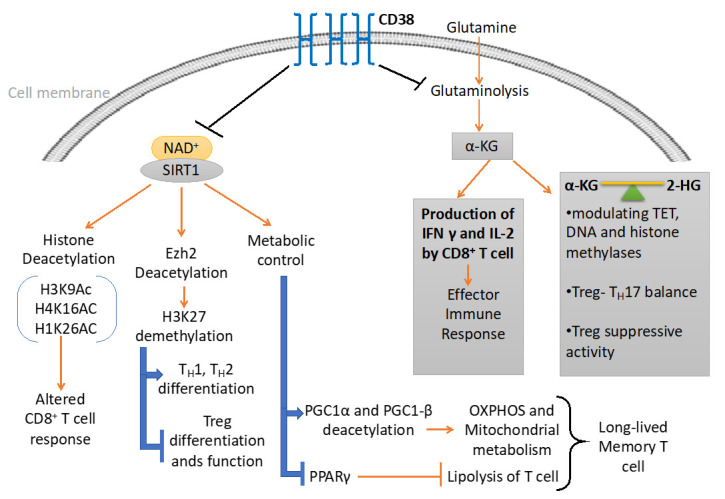
CD38 mediated regulation of metabolic pathways and chromatin modifications in T cells. CD38 affects the differentiation and effector response of T cells through modulating the metabolic pathways and epigenetic landscape of T cells. On one hand, CD38 curtails the availability of NAD^+^ to Sirt1 and hence attenuates its enzymatic activity which regulates different metabolic and epigenetic pathways in T cells. On the other hand, CD38 inversely regulates glutaminolysis pathways, which not only regulate effector cytokine production in T cells but also produce α-ketoglutarate (α-KG) and 2-hydroxyglutarate (2-HG), important epigenetic modifiers.

NAD^+^-Sirt1 axis, which is regulated by the expression of CD38 plays a vital role in determining the metabolic commitment of T cells. Importantly, Sirt1 has shown to promote oxidative phosphorylation and mitochondrial metabolism [124,125,126,127], which has appeared to be crucial in the differentiation of memory T cell [128,129]. Sirt1 mediated regulation of mitochondrial metabolism is mainly attributed to proteins belonging to peroxisome proliferators γ co-activator 1 (PGC-1) family, PGC1α and PGC1β [127,130]. The activity of PGC1α and PGC1β are known to be promoted via Sirt1 mediated deacetylation. The activation of these proteins facilitates mitochondrial biogenesis and subsequently OXPHOS [130]. Thus, it might be hypothesized that hyperactivity of CD38 alleviates Sirt1 activity and hence perturb mitochondrial biogenesis and OXPHOS, which impinge on memory T cell differentiation. It should also be noted that Sirt1 could promote the differentiation of long-lived T cells with heightened anti-tumor potential through inhibiting the activity of PPARγ [131,132], which suppresses the induction of lipolysis in T cells [132], a prerequisite for memory T cell differentiation [132,133]. Therefore, it seems that the expression of CD38 on T cells by limiting intracellular NAD^+^ levels and Sirt1 activity exerts metabolic perturbations, which ultimately affects T cell differentiation and functionality. Further investigation is thus needed to delineate the intricate mechanisms which would be useful in devising drugable target to improve the metabolic fitness and hence the functionality of T cells (Figure 2).

## 6. CD38-NAD^+^ Axis and T Cell Epigenetic Modifications

CD38, by virtue of its NADase activity, has shown to perturb cellular homeostasis of different NAD^+^ consuming enzymes reported to act as epigenetic modifiers and hence can alter the functional fate of T cells [134]. Emerging evidence suggests that CD38-NAD^+^ axis has a profound influence in regulating the intracellular levels of various metabolites including α-ketoglutarate (α-KG), 2-hydroxyglutarate (2-HG), and signaling mediator like ROS, which are reported to play a pivotal role in orchestrating the epigenetic landscape of T cells [13,135,136]. Thus, a detailed discussion of these pathways is of utmost importance in the aspect of T cell differentiation, development, and function.

### 6.1. Metabolites Mediated Epigenetic Regulation

As discussed in the previous section, elevated expression of CD38 on T cells inversely regulates glutaminolysis, a predominant pathway of yielding α-KG, which act as a co-factor of histone and DNA demethylases [13,135]. Recently, it has been reported that α-KG mediated H3K27 demethylation can be linked to the increased effector cytokines (IFN-γ and IL-2) production by mouse CD8^+^ T cell [137]. The study arouses the possible association of CD38 dependent metabolic rewiring as a critical cellular event regulating the epigenetic modification of T cells and hence their functional state. This is supported by the recent studies showing that expression of CD38 facilitates T cell exhaustion at the tumor site, which is refractory to restore their functionality by immune checkpoint blockade therapy [52]. This phenomenon is in part due to the extensive epigenetic remodeling of CD38 expressing stable exhausted T cells (CD38^+^PD1^+^CD101^+^ T cells) [52]. Although the detailed mechanisms underpinning CD38 dependent epigenetic modification of T cells has not been fully explored, altered metabolic commitment of CD38 expressing T cells could play an essential role in this process.

Several studies have reported that a balance between intracellular level of α-KG and 2-HG, a metabolite produced by isocitrate dehydrogenase 1 and 2 (IDH1/2), are capable of altering histone methylation and chromatin accessibility in various cell types [135,138]. α-KG and 2-HG are mutually antagonistic in nature and are found to modulate epigenetic modification of T cells via affecting the activity of ten-eleven Translocases (TET), DNA, and histone methylases [135]. The intricate balance between α-KG and 2-HG in instilling epigenetic modification has recently been implicated in fate determination of Th17 and Treg [138]. This is in accordance with the early observation showing that glutaminolysis derived α-KG negatively regulates Treg differentiation [139]. The mechanism could be of further importance in explaining the elevated expression of CD38, particularly on Treg with heightened suppressive activity [42]. It is possible that CD38 mediated negative regulation of glutaminolysis, and hence the production of α-KG and subsequently 2-HG, maintains demethylation state of *FoxP3* promotor that results in increased Treg stability.

### 6.2. Sirt1 Dependent Epigenetic Regulation

NAD^+^-Sirt1 axis, which is inversely regulated by CD38 expression, has been reported to be an important epigenetic modifier owing to its deacetylation activity [140]. The Sirt1 induced epigenetic regulation can be achieved in three distinct mechanisms, viz., (a) regulation of chromatin structure by histone deacetylation, (b) regulating the activity of transcription factor by deacetylation, and (c) regulation of other epigenetic enzymes by deacetylation [140].

Sirt1 can deacetylate lysine residues of different histone marks, including H3K9Ac, H4K16Ac, and H1K26Ac, as silencing Sirt1 using RNAi approach in human cells led to a global increase in H3K9Ac and H4K16Ac [141]. In human CD8 memory T cells, increased histone acetylation at H3K9 (H3K9Ac), is associated with the activate transcription of *EOMES*, *PRF1*, and *GZMB* loci [142]. However, whether Sirt1 has any role in regulating the acetylation of H3K9 at *EOMES*, *PRF1*, and *GZMB* loci of memory CD8 T cells has not been fully explored and thus warrants further investigations.

The role of Sirt1 on imparting distinctive epigenetic signature on T cells could also be mediated through regulating the activity of epigenetic enzymes. It has recently been demonstrated that CD38 ablation mediated elevation of Sirt1 in CD8 T cells from SLE patients is capable of deacetylating enhancer of zeste homolog 2 (Ezh2), an enzyme catalyzing methylation of H3K27 which ultimately causes transcriptional repression [143]. The study further reported that Sirt1 mediated deacetylation of Ezh2 rendered it inactive, resulting in increased transcription of *T-bet, EOMES,* and *Runx3* in CD8 T cells due to reduced Ezh2 mediated H3K27me3 in these gene loci [143]. The study, thus, pointed out the role of CD38-NAD^+^-Sirt1 axis mediated regulation of Ezh2 in determining the cytotoxic potential of CD8 T cells. Ezh2 mediated H3K27 tri-methylation is also reported to regulate Th1, Th2 and Treg differentiation. Ezh2 and increased H3K27 tri-methylation inhibits Th1 and Th2 differentiation as it facilitates the silencing of genes encoding lineage-specific cytokines (like *Ifng* and *il13* for Th1 and Th2, respectively) and transcription factors (*T-bet* and *GATA3* for Th1 and Th2, respectively) [144].

Conversely, expression of Ezh2 promotes Treg cell stability and function, as genetic ablation of *Ezh2*, specifically in FoxP3 expressing T cells, has shown to suppress Treg cell signature gene FoxP3 [145]. From a recent clinical study, it was found that T cells from Rheumatoid arthritis (RA) patients exhibited lower Ezh2, which regulated T cell differentiation through promoting epigenetic modification [146]. From in vitro studies, it was concluded that attenuation of Ezh2 led to the downregulation of RUNX1, and promoted SMAD7 which synergistically dampened the TGFβ signaling events, essential for generation of Tregs [146]. Therefore, it seems reasonable to argue that in addition to directly influencing FoxP3 activity and stability, Sirt1 could indirectly affect the fate of Treg via regulating the enzymatic activity of Ezh2 [112,113] (Figure 2).

## 7. Conclusions

From the above discussion, the multifaceted roles of CD38 in T cell differentiation, development, and different aspects of T cell health is quite evident. In addition to controlling the different aspects of T cell activation by interplaying with the TCR downstream signaling pathways, the competition of CD38 with several post-translational and epigenetic modifiers for occupancy of NAD^+^ has been shown to be one of the key dictating factors driving discrete T cell fates. Even this intricate balance appears to be decisive in regulating the suppressive potential of Treg. It can be speculated from the existing studies that Sirt1 mediated deacetylation of FoxP3, a post-translational event that diminishes the suppressive potential of Treg could be instrumental in regulating the differential suppressive activity between CD38^hi^ and CD38^lo^ Treg. In addition to Sirt1 axis, CD38 mediated metabolic rewiring could also play a crucial role in this context through orchestrating the cellular balance of α-KG and 2-HG; key glutaminolysis derived metabolites have shown to regulate epigenetic modification. Therefore, it seems that although CD38 expression during activation of T cells might be necessary for mediating early activation events, its stabilization could have a differential effect in defining the functional outcome of different T cell subsets.

It is also evident from recent studies that CD38 has a crucial role in driving stable exhaustion of T cells, which is refractory to the PD-1 mediated functional rejuvenation. Although the precise mechanism(s) yet to decipher, it seems that epigenetic modification could play a pivotal role in inducing the stable exhausted phenotype of CD38^hi^ T cells. The notion can be supported by the fact that decreased deacetylase activity of Sirt1 in CD38^hi^ T cells attenuates the enzymatic activity of histone methyltransferase Ezh2 which has a profound effect in determining the functionality and survival of T cells. Depleting CD38 levels in these cells by administration of an antibody against CD38 along with immune checkpoint blockade (anti-PD1) could potentially rejuvenate these cells from being exhausted. These could result in a better manifestation of the anti-tumor property of the tumor-infiltrating T cells in the advanced stage of a tumor, and in the resolution of any chronic infections, which also induce stable exhaustion phenotype of T cells. However, it should be noted in this context that CD38 antibodies which are available for clinical evaluation including Daratumumab, Isatuximab, MOR202, and TAK-079, cause depletion of target cells through multiple mechanisms including antibody dependent cellular cytotoxicity (ADCC), complement dependent cytotoxicity (CDC), antibody dependent cellular phagocytosis (ADCP), etc. [27,147]. Therefore, clinical use of these antibodies to restore the functionality of CD38^+^ exhausted T cells in solid tumors or other chorionic infections might cause an adverse effect due to depletion of T cells. Considering this fact, prudent selection of CD38 antibodies specifically targeting the NADase/glycohydrolase activity without triggering target cell cytotoxicity would be extremely important to garner T cell mediated tumor killing and exploiting this strategy for improved clinical outcomes in solid tumors. The future of CD38 research thus far has much vital information to offer, unraveling some novel downstream mechanisms, and this could emerge as one of the principal pharmacological tools in the hands of the scientific and medical fraternity to modulate functionality T cells in varied disease scenario.

## Abbreviations

NAD	Nicotinamide Adenine Dinucleotide
NADP	Nicotinamide Adenine Dinucleotide Phosphate
NAADP	Nicotinic Acid Adenine Dinucleotide Phosphate
ADPR	Adenosine Di-phosphate Ribose
cADPR	cyclic Adenosine Di-phosphate Ribose
TCR	T Cell Receptor
PD1	Programmed Death 1
CTLA4	Cytotoxic T-Lymphocyte-Associated protein 4
Lag3	Lymphocyte Activation Gene-3
Tim3	T-cell Immunoglobulin domain and Mucin domain 3
ATP	Adenosine Tri-Phosphate
ADP	Adenosine Di-Phosphate
AMP	Adenosine Mono-Phosphate
MM	Multiple Myeloma
DC	Dendritic Cell
MDSC	Myeloid Derived Suppressor Cells
Treg	Regulatory T cells
CRC	Colo-Rectal Cancer
SOCE	Store-Operated Calcium Entry
CRAC	Calcium Release-Activated Channel
IP3	Inositol Tri-Phosphate
IP3R	Inositol Tri-Phosphate Receptor
ER	Endoplasmic Reticulum
RyR	Ryanodine Receptor
SERCA	Sarcoendoplasmic Reticulum Ca^2+^ ATPase
M. avium	Mycobacterium avium
IL	Interleukin
IFN-γ	Interferon γ
GM-CSF	Granulocyte Macrophage Colony Stimulating Factor
NFAT	Nuclear factor of activated T-cells
NFATc1	Nuclear factor of activated T-cells cytoplasmic 1
NFATc2	Nuclear factor of activated T-cells cytoplasmic 2
ChIP	Chromatin Immunoprecipitation
MAPK	Mitogen Activated Protein Kinase
PTK	Protein Tyrosine Kinase
ZAP70	Zeta-chain-associated protein kinase 70
PLCg1	Phospho-Lipase C Gamma 1
PARP	Poly-ADP Ribose Polymerase
ART	ADP Ribosyl Transferase
SIRT	Sirtuin
LEF-1	Lymphoid enhancer-binding factor 1
NICD	NAD^+^-induced cell death
P2RX7	P2X purinoceptor 7
R35	Arginine 35
STAT	Signal Transducer and Activator of Transcription
TNFα	Tumor Necrosis Factor α
NF-kB	Nuclear Factor kappa-light-chain-enhancer of Activated B cells
FoxP3	Forkhead box Protein3
CNS2	Conserved Non-coding DNA Sequence 2
HDAC	Histone Deacetylase
EAE	Experimental Autoimmune Encephalomyelitis
Bclaf1	BCL2 Associated Transcription Factor 1
SLE	Systemic Lupus Erythematosus
RORγ	RAR-related Orphan Receptor Gamma
OXPHOS	Oxidative Phosphorylation
PGC1	Peroxisome Proliferators γ Co-activator 1
PPARγ	Peroxisome P*roliferator-activated* R*eceptor* G*amma*
α-KG	α Keto Gluterate
2-HG	2-Hydroxy Gluterate
IDH1/2	Isocitrate Dehydrogenase1/2
EZH2	Enhancer of Zeste Homolog 2
RA	Rheumatoid Arthritis
